# Outcome and Predictors for Mortality in Patients with Cardiogenic Shock: A Dutch Nationwide Registry-Based Study of 75,407 Patients with Acute Coronary Syndrome Treated by PCI

**DOI:** 10.3390/jcm10102047

**Published:** 2021-05-11

**Authors:** Mina Karami, Elma J. Peters, Wim K. Lagrand, Saskia Houterman, Corstiaan A. den Uil, Annemarie E. Engström, Luuk C. Otterspoor, Jan Paul Ottevanger, Irlando A. Ferreira, Jose M. Montero-Cabezas, Krischan Sjauw, Jan van Ramshorst, Adriaan O. Kraaijeveld, Niels J. W. Verouden, Erik Lipsic, Alexander P. Vlaar, Jose P. S. Henriques, on Behalf of the PCI Registration Committee of The Netherlands Heart Registration

**Affiliations:** 1 Heart Center, Department of Cardiology, Amsterdam Cardiovascular Sciences, Amsterdam UMC, University of Amsterdam, 1105 AZ Amsterdam, The Netherlands; minakarami@amsterdamumc.nl (M.K.); e.j.peters@amsterdamumc.nl (E.J.P.); 2 Department of Intensive Care Medicine, Amsterdam UMC, University of Amsterdam, 1105 AZ Amsterdam, The Netherlands; w.k.lagarand@amsterdamumc.nl (W.K.L.); a.p.vlaar@amsterdamumc.nl (A.P.V.); 3 Netherlands Heart Registration, 3511 EP Utrecht, The Netherlands; saskia.houterman@nederlandsehartregistratie.nl; 4 Department of Intensive Care Medicine, Erasmus MC, 3015 GD Rotterdam, The Netherlands; c.denuil@erasmusmc.nl; 5 Department of Intensive Care Medicine, Maasstad Hospital, 3079 DZ Rotterdam, The Netherlands; 6 Department of Intensive Care Medicine, Franciscus Gasthuis, 3004 BA Rotterdam, The Netherlands; a.engstrom@franciscus.nl; 7 Department of Cardiology, Catherina Hospital, 5623 EJ Eindhoven, The Netherlands; otterspoor@hotmail.com; 8 Department of Cardiology, Isala Hospital, 8025 AB Zwolle, The Netherlands; j.ottevanger@isala.nl (J.P.O.); i.andradeferreira@isala.nl (I.A.F.); 9 Department of Cardiology, Leiden University Medical Center, Leiden University, 2333 ZA Leiden, The Netherlands; J.M.Montero_Cabezas@lumc.nl; 10 Department of Cardiology, Medical Center Leeuwarden, 8934 AD Leeuwarden, The Netherlands; k.d.sjauw@gmail.com; 11 Department of Cardiology, Noordwest Hospital Group, 1815 JD Alkmaar, The Netherlands; J.van.Ramshorst@nwz.nl; 12 Department of Cardiology, UMC Utrecht, 3584 CX Utrecht, The Netherlands; a.o.kraaijeveld-3@umcutrecht.nl; 13 Department of Cardiology, Amsterdam Cardiovascular Sciences, Amsterdam UMC, Vrije Universiteit Amsterdam, 1081 HV Amsterdam, The Netherlands; c.verouden@amsterdamumc.nl; 14 Department of Cardiology, University Medical Center Groningen, 9713 GZ Groningen, The Netherlands; e.lipsic@umcg.nl

**Keywords:** cardiogenic shock, percutaneous coronary intervention, acute coronary syndrome, clinical outcome, predictors

## Abstract

It is important to gain more insight into the cardiogenic shock (CS) population, as currently, little is known on how to improve outcomes. Therefore, we assessed clinical outcome in acute coronary syndrome (ACS) patients treated by percutaneous coronary intervention (PCI) with and without CS at admission. Furthermore, the incidence of CS and predictors for mortality in CS patients were evaluated. The Netherlands Heart Registration (NHR) is a nationwide registry on all cardiac interventions. We used NHR data of ACS patients treated with PCI between 2015 and 2019. Among 75,407 ACS patients treated with PCI, 3028 patients (4.1%) were identified with CS, respectively 4.3%, 3.9%, 3.5%, and 4.3% per year. Factors associated with mortality in CS were age (HR 1.02, 95%CI 1.02–1.03), eGFR (HR 0.98, 95%CI 0.98–0.99), diabetes mellitus (DM) (HR 1.25, 95%CI 1.08–1.45), multivessel disease (HR 1.22, 95%CI 1.06–1.39), prior myocardial infarction (MI) (HR 1.24, 95%CI 1.06–1.45), and out-of-hospital cardiac arrest (OHCA) (HR 1.71, 95%CI 1.50–1.94). In conclusion, in this Dutch nationwide registry-based study of ACS patients treated by PCI, the incidence of CS was 4.1% over the 4-year study period. Predictors for mortality in CS were higher age, renal insufficiency, presence of DM, multivessel disease, prior MI, and OHCA.

## 1. Introduction

The mortality rate of patients with acute coronary syndrome (ACS) has declined rapidly in recent years due to preventive measures (e.g., cholesterol reduction) and advanced treatment strategies (e.g., revascularization) [1]. However, for patients with acute myocardial infarction (MI) who develop cardiogenic shock (CS), mortality remains unacceptably high at around 50% [2]. Based on the demographics of the Dutch population, which resembles the global trend in population aging, an increase in the incidence of acute MI is expected within the next 20 years [3]. As around 5–10% of patients with acute MI develop CS, management of this clinically challenging population will become an even more important health problem worldwide.

It is essential to gain more insight into CS patients, as currently, little is known on the best treatment strategy to improve outcome. Furthermore, if we can determine prognostic characteristics in these patients, we may be able to identify patients that are at greater risk of death and develop preventive measures and patient-specific treatment strategies.

By studying an unselected large and complete real-world cohort of patients treated with percutaneous coronary intervention (PCI) registered prospectively in the nationwide Netherlands Heart Registration (NHR), the aim of this study was to assess the incidence, clinical outcome, and predictors of mortality for CS in ACS patients over a time period of 4 years.

## 2. Materials and Methods

### 2.1. Study Design

The NHR is a Dutch nationwide registry on all cardiac interventions and surgical procedures, comprising data of 73 centers (PCI or heart center *n* = 30). The NHR main objectives are to maintain and improve quality of care by registering, analyzing, and providing relevant information on treatment of cardiac disease. The NHR registers clinical characteristics and outcome of patients with cardiac disease. Data collection and registration is performed by the participating centers in a secured online environment. A waiver for consent for the NHR data registry was obtained from the Medical Ethics Committee. The study protocol conforms to the ethical guidelines of the 1975 Declaration of Helsinki. For the purpose of this study, NHR data were extracted on patients treated with PCI from the registry inception in the year 2015 until 2019. We performed our analysis on patients treated with PCI for the indication ACS.

### 2.2. Definitions

CS was registered in the NHR database if present at admission for PCI. CS was defined as the presence of hypotension (systolic blood pressure (SBP) ≤ 90 mmHg during ≥30 min, or hemodynamic support required to maintain a SBP ≥ 90 mmHg), together with clinical signs of hypoperfusion (i.e., cold extremities, oliguria <30 mL/h, and/or a heart rate ≥60 beats per minute). ACS was defined as a ST-segment elevation MI (STEMI) or non-STEMI (NSTEMI). MI was defined according to the Third Universal Definition of MI [4]. STEMI was defined as acute chest pain in the presence of ST-segment elevation longer than 20 min. NSTEMI was defined as acute chest pain without the presence of ST-segment elevation, including unstable angina pectoris. There was a universal approach with direct coronary angiography in cases of NSTEMI. Out-of-hospital cardiac arrest (OHCA) was defined as a cardiac arrest that involved defibrillation with or without cardiopulmonary resuscitation (CPR), which occurred in the prehospital setting before and in relation to the PCI indication. Multivessel PCI was defined as more than one vessel treated during intervention. The culprit vessel was defined as the vessel that was registered as the primary treated vessel during PCI. Chronic total occlusion (CTO) was defined as the presence of an atherosclerotic occlusion for more than 3 months and Thrombolysis In Myocardial Infarction (TIMI) flow grade of 0 or 1 in one of the treated coronary arteries. Multivessel disease was defined as the presence of a stenosis of >70% in luminal diameter in more than two native major coronary arteries or first-order side branches. Dialysis was defined as hemodialysis, peritoneal dialysis, or continuous veno-venous hemofiltration for renal failure, present at admission for PCI. Serum creatinine level was measured up to 3 months prior to the PCI or on the date of intervention. Estimated glomerular filtration rate (eGFR) in mL/min/1.73 m^2^ was calculated by the following formula: 175 × ((serum creatinine level/88.4) − 1.154) × (age − 0.203) × (0.742 if female). Left ventricular ejection fraction (LVEF) was measured up to 6 months prior to the intervention. Descriptive LVEF data were converted into a percentage according to: good LVEF 55%, moderate 40%, poor 25%, and severe 20%. Clinical outcome measures were: 30-day and 1-year mortality from PCI date, urgent coronary bypass grafting (CABG) within 24 h after PCI, MI within 30 days after PCI (including STEMI and NSTEMI, excluding periprocedural MI (i.e., type 4; occurring within 48 h after PCI)) and target vessel revascularization (TVR) within 1 year after PCI (defined as revascularization by PCI in the index coronary artery).

### 2.3. Statistical Analysis

The primary outcome of this study was mortality for patients with CS compared to patients without CS. Survival curves were displayed as Kaplan–Meier curves and compared with log-rank test. Furthermore, median follow-up with interquartile ranges (IQR) was calculated for both groups using time (in months) between the date of intervention and last follow-up or death. Secondary outcomes were (1) the incidence of CS, (2) predictors for mortality in patients with CS, and (3) the difference in characteristics and clinical outcome in patients with and without CS. All data were analyzed per patient, not per registered PCI. If a patient had multiple PCIs, we selected the first intervention in which CS was present (shock cohort). In patients without the presence of CS at admission for PCIs, we selected the first intervention that was performed in the patient (no-shock cohort). The incidence of CS per year was calculated as the number of patients with the condition divided by the total number of patients treated with PCI. A Cox proportional-hazard regression analysis was used to identify predictors of mortality in patients with CS. The dependent variable was mortality, and the independent variables were all patient characteristics reported in Table 1 (with less than 20% missing values), including type of treatment center (PCI or heart center) and the year of intervention. A stepwise method (using backwards elimination) and enter method were compared before selection of variables for the final model. For the enter method, variables with a *p*-value < 0.10 in univariable analysis were included in the multivariable model. The association between the dependent and independent variables was described as a hazard ratio (HR) with a corresponding 95% confidence interval (CI). The non-linearity of variables was tested by categorization into quartiles. A receiver operating characteristic (ROC) curve was used to illustrate the performance of classification according to the multivariable model and an area under the ROC curve (AUC) was calculated. To assess the association between factors and longer-term mortality (after 30 days), we also performed a landmark analysis excluding patients who had died before or at 30 days, using univariable and multivariable Cox regression analysis. 

In addition, the differences in characteristics and clinical outcome between patients with and without CS before PCI were compared. Normally distributed data were described as mean ± standard deviation (SD) and compared with the t-test. Not-normally distributed data were described as median with IQR and compared using the Mann–Whitney U test. Categorical data were described as frequencies with percentages and compared using the Fisher’s exact or Chi-square test, whichever appropriate. A *p*-value < 0.05 was considered statistically significant for all non-specified analyses. Statistical analyses were performed using IBM SPSS Statistics version 26.0.

## 3. Results

### 3.1. Study Population

**Figure 1:**
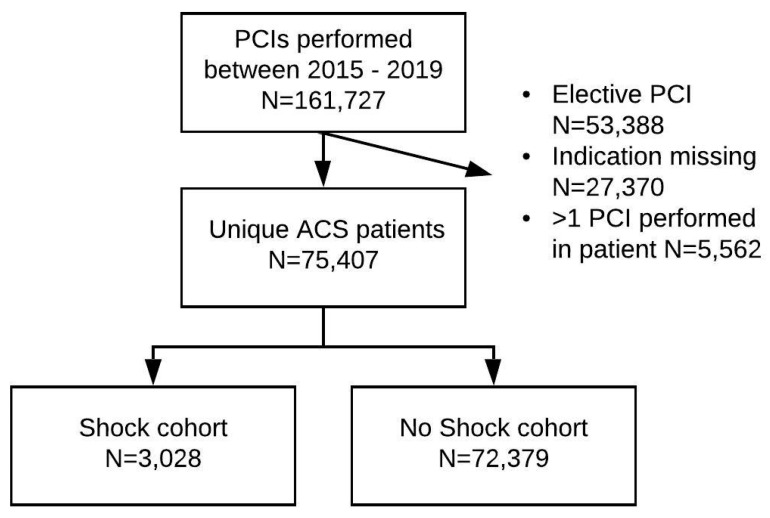
Flowchart of study population: acute coronary syndrome patients treated by percutaneous coronary intervention with (*n* = 3028) and without cardiogenic shock (*n* = 72,379).

In the time period 2015 until 2019, 80,969 PCIs were performed in the Netherlands for the indication ACS in 75,407 patients (Figure 1: flowchart). Among ACS patients who were treated with PCI, 3028 patients (4.1%) were identified with CS. The incidences of CS in the 4-year period of this study were respectively 4.3%, 3.9%, 3.5%, and 4.3% per year. 

### 3.2. Baseline Characteristics 

Clinical and procedural characteristics are presented in Table 1. Patients with and without CS were similar in percentage of males (71% versus 72%, *p* = 0.74) and presence of diabetes mellitus (DM) (18% versus 19%, *p* = 0.41). Patients with CS were slightly older (66 ± 12 years versus 65 ± 12 years, *p* < 0.001), more frequently had multivessel disease (59% versus 46%, *p* < 0.001), presence of CTO (3% versus 2%, *p* < 0.01), OHCA (45% versus 4%, *p* < 0.001), and worse renal function (eGFR category < 15; 2% versus 0.7%, eGFR 15–29; 5% versus 2%, eGFR 30–59; 38% versus 19% and eGFR ≥ 60; 56% versus 79%, *p* < 0.001), compared to patients without CS. In addition, CS patients less frequently had prior MI (17% versus 19%, *p* = 0.02) and prior CABG (5% versus 7%, *p* < 0.001). Moreover, patients with CS had worse LVEF (*p* < 0.001), more often dialysis (*p* = 0.03), and less prior PCI (*p* < 0.001), but these findings are limited due to the amount of missing data (42–69%). Of all ACS patients who underwent PCI, 66% was treated in a heart center and 35% in a PCI center. The indication for PCI differed between patients with and without CS (*p* < 0.001). In CS patients, the indication for PCI was STEMI in 89% and NSTEMI in 11%. In patients without CS, the PCI indication was STEMI in 46% and NSTEMI in 54%. The first access method for performing PCI in patients with CS was via the radial artery in 50% and femoral artery in 50%. In patients without CS, PCI was performed via the radial artery in 84% and the femoral artery in 15% of the cases. Data were missing in 50% of the cases on culprit lesion location and whether culprit lesion or multivessel PCI was performed. 

### 3.3. Clinical Outcome 

**Figure 2:**
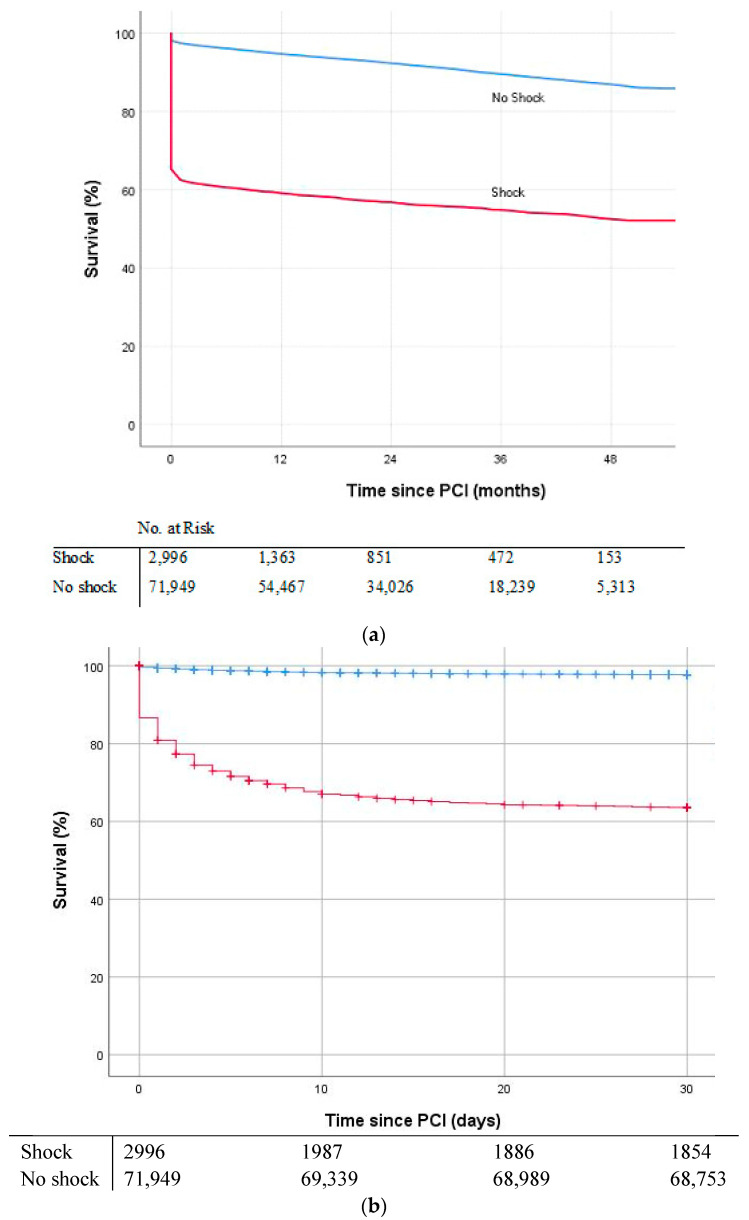
Kaplan–Meier curves showing survival of acute coronary syndrome patients treated by percutaneous coronary intervention with and without cardiogenic shock at admission. (**a**) Over the 4-year study period, (**b**) within 30-days.

Clinical outcomes are presented in Table 1. Median duration of follow-up was 9 months (0–26) for patients with CS and 22 months (12–36) for patients without CS. Thirty-day mortality was 36% for CS patients versus 2% for patients without CS, *p* < 0.001. One-year mortality was 40% for CS patients versus 5% for patients without CS, *p* < 0.001. Figure 2a,b show the Kaplan–Meier curves for survival over the 4-year study period and within 30-days (both, log-rank *p* < 0.001). Patients with CS more often required an urgent CABG within 1 day (1% versus 0.2%, *p* < 0.001) and more often experienced MI within 30 days after PCI (1% versus 0.7%, *p* < 0.01), compared to patients without CS.

### 3.4. Predictors for Mortality in Cardiogenic Shock

The results of the Cox regression analysis are shown in Table 2. Factors that were identified in multivariable analysis as independent predictors for mortality in patients with CS were higher age (HR 1.02, 95%CI 1.02–1.03, *p* < 0.001), lower eGFR (HR 0.98, 95%CI 0.98–0.99, *p* < 0.001), presence of DM (HR 1.25, 95%CI 1.08–1.45, *p* < 0.01), multivessel disease (HR 1.22, 95%CI 1.06–1.39, *p* < 0.01), prior MI (HR 1.24, 95%CI 1.06–1.45, *p* < 0.01), and OHCA (HR 1.71, 95%CI 1.50–1.94, *p* < 0.001). The enter and stepwise method resulted in similar multivariable models.

The ROC curve of classification according to the multivariable model is shown in the Supplementary Materials, Figure S1. At internal validation, the model had an acceptable performance, with an AUC of 0.73. Landmark analysis after exclusion of patients who had died before or at 30 days is presented in the Supplementary Materials Table S1. In the multivariable Cox regression analysis, OHCA was the only factor that was no longer a predictor for mortality. 

Furthermore, in the Supplementary Materials, Kaplan–Meier survival curves are shown for patients with CS according to different age categories (Figure S2), renal function (Figure S3), and OHCA vs. no OHCA (Figure S4).

## 4. Discussion

In this large contemporary cohort of ACS patients treated with PCI in the Netherlands, the incidence of CS was 3.5–4.3% per year within a time period of 4 years. CS patients had a poor survival compared to patients without CS, and their survival rate did not significantly improve over the 4-year time period. 

The AMIS registry of ACS patients in Switzerland found that the incidence of CS decreased between the years 1997 and 2017, from 8.7% (period 1997–2006) to 7.3% (2007–2017) [5]. During this time, the incidence of CS that developed during hospital stay declined from 7.8% to 3.5%, but the incidence of CS on admission increased from 2.5% to 4.6%. The authors speculate that the decline of CS during hospital stay may be due to improvements in the medical treatment, increase of PCIs (causing reduction of the infarct size), and earlier arrival at the hospital. The increase of CS at admission on the other hand may be due to the more frequent and rapid transportation of sicker patients who would otherwise have died before hospital arrival. The 4.6% incidence from the AMIS registry in the time period 2007–2017 corresponds with the 4.1% incidence of CS on admission in our Dutch population during the years 2015–2018. Accordingly, the Swedeheart registry, which collects data on patients who underwent coronary angiography or were treated by PCI in Sweden, found a 4% incidence for CS complicating AMI in the year 2012 [6].

Identifying CS patients who have an increased risk of death is important, since risk stratification can help us select patients for therapies such as mechanical circulatory support. These prognostic characteristics may also be used to reduce treatment selection bias in studies or the comparison of outcomes between different centers. There are several existing risk scores designed to predict outcome in CS patients. For example, the CardShock score includes the following risk factors in their calculation: age > 75 years, eGFR, prior MI/CABG, confusion, lactate, CS etiology, and LVEF [7]. The IABP-SHOCK score includes age > 73 years, glucose level >10.6 mmol, creatinine, lactate, and TIMI flow <3 after PCI [8]. In our study, mortality in CS patients was mainly driven by a higher age, renal insufficiency, and OHCA. This finding emphasizes the importance of including these characteristics in risk stratification. In accordance with characteristics included in current risk scores, we also found an association between increased mortality and prior MI, presence of DM, and multivessel disease. The performance of the multivariable model for predicting mortality in CS patients was acceptable (AUC = 0.73). Sex-related differences were not associated with survival after multivariable adjustment and probably based on the difference in age and comorbidities between males and females.

The mortality of patients with CS is mostly determined in the acute phase. Our results showed that the mortality of CS patients after 30 days was almost identical to patients without CS. Additional mortality after 30 days was 4% in CS and 3% for patients without CS. In the landmark analysis of patients who survived the first 30 days, there was no longer an association between OHCA and mortality. Therefore, OHCA-driven mortality is probably only evident in the acute phase, while other factors are associated with both short-term and longer-term mortality. 

The Swedeheart registry reported that STEMI patients more often developed CS, compared to NSTEMI patients (*p* < 0.001) [6]. Similarly, we found that STEMI patients more often presented with CS than NSTEMI patients. Several studies found that although STEMI patients had a higher in-hospital and short-term mortality compared with NSTEMI patients, long-term mortality was similar [9,10]. In our study, in patients with CS, STEMI was associated with increased mortality in univariable Cox regression analysis, but this was not significant after multivariable adjustment. 

The strength of our study is that it consists of a large nationwide cohort and represents real-world outcomes. The data are contemporary, as they reflect patients who were treated in recent years by PCI. It is the first study to report outcomes specifically for ACS patients treated by PCI with and without CS in the Netherlands. A limitation of our study is that although it represents an unselected cohort, only patients treated with PCI are included in the registry. Patients who died prehospital or before the procedure are not registered, and therefore, the outcomes for CS may be even worse than reported in this study. Furthermore, the NHR is the first nationwide registry on cardiac interventions, which was developed relatively recently in 2015 and it is still being further expanded. A limitation of the registry is that currently, only data are collected on CS that are present at admission, and no data are available on the development of CS after leaving the catheterization laboratory. In addition, the completeness and accuracy of data input is a limitation. A rather large amount of data regarding important variables such as LVEF and culprit lesion location were missing, and not all data that are of clinical and scientific interest are currently collected in the registry (e.g., use of medication, use of mechanical support devices, hemodynamic parameters such as blood pressure, duration of cardiac arrest, laboratory values such as lactate, and success of intervention). This also hampered the calculation of validated risk scores such as IABP-SHOCK II and CardShock). Finally, this study reflects the clinical practice in the Netherlands and may not be representative for other countries. Local differences in the distribution of heart and PCI centers, organization of emergency medical services, and patient management may result in different outcomes. 

## 5. Conclusions

In this Dutch nationwide registry-based study on ACS patients treated with PCI, CS had an incidence of 4.1% and was associated with an increased mortality. The survival of CS patients did not improve over the 4-year time period. Factors associated with worse survival in patients with CS were higher age, lower eGFR, presence of DM, multivessel disease, prior MI, and OHCA.

## Figures and Tables

**Table 1:** Characteristics and clinical outcome of acute coronary syndrome patients treated by percutaneous coronary intervention with and without cardiogenic shock at admission.

	All Patients	Missing	Shock	No Shock	*p*-Value
	(*n* = 75,407)		(*n* = 3028)	(*n* = 72,379)	
**Clinical characteristics**					
Age (years)	65 ± 12	0 (-)	66 ± 12	65 ± 12	<0.001
Male	53,945 (72)	0 (-)	2158 (71)	51,787 (72)	0.74
Diabetes mellitus	13,957 (19)	2084 (3)	522 (18)	13,435 (19)	0.41
Dialysis	172 (0.5)	42,701 (57)	13 (0.9)	159 (0.5)	0.03
Multivessel disease	34,781 (46)	523 (0.7)	1770 (59)	33,011 (46)	<0.001
Chronic total occlusion	1657 (2)	1245 (2)	90 (3)	1567 (2)	<0.01
Prior myocardial infarction	13,588 (19)	2137 (3)	484 (17)	13,104 (19)	0.02
Prior PCI	8352 (19)	31,815 (42)	282 (15)	8067 (19)	<0.001
Prior CABG	5136 (7)	1162 (2)	149 (5)	4989 (7)	<0.001
Out of hospital cardiac arrest	4112 (5)	94 (0.1)	1373 (45)	2739 (4)	<0.001
Renal function (mL/min/1.73 m^2^)		8207 (11)			<0.001
eGFR ≥60	52,257 (78)		1485 (56)	50,772 (79)	
eGFR 30–59	13,243 (20)		1002 (38)	12,241 (19)	
eGFR 15–29	1205 (2)		130 (5)	1075 (2)	
eGFR <15	495 (0.7)		45 (2)	450 (0.7)	
LVEF		51,820 (69)			<0.001
>50%	14,087 (60)		161 (22)	13,926 (61)	
30–50%	7562 (32)		319 (44)	7243 (32)	
≤30%	1938 (8)		241 (33)	1697 (7)	
**Treatment center**		0 (-)			<0.001
Heart center	49,396 (66)		2133 (70)	47,263 (65)	
PCI center	26,011 (35)		895 (30)	25,116 (35)	
**Procedure characteristics**					
PCI indication		0 (-)			<0.001
STEMI	36,288 (48)		2704 (89)	33,584 (46)	
NSTEMI	39,119 (52)		324 (11)	38,795 (54)	
Culprit lesion		37,340 (50)			0.02
LAD	15,425 (41)		619 (38)	14,806 (41)	
Other	22,642 (60)		1018 (62)	21,624 (60)	
PCI access method (1st)		41,448 (55)			<0.001
Radial	28,172 (83)		671 (50)	27,501 (84)	
Femoral	5704 (17)		681 (50)	5023 (15)	
Brachial	83 (0.2)		4 (0.3)	79 (0.2)	
Culprit lesion PCI	25,477 (67)	37,327 (50)	1034 (63)	24,443 (67)	<0.01
Multivessel PCI	12,603 (33)	37,327 (50)	603 (37)	12,000 (33)	<0.01
**Clinical outcome**					
30-day mortality	2722 (4)	462 (0.6)	1080 (36)	1642 (2)	<0.001
1-year mortality *	3346 (6)	228 (0.4)	855 (40)	2491 (5)	<0.001
Urgent CABG within 1 day	206 (0.3)	2002 (3)	38 (1)	168 (0.2)	<0.001
MI within 30 days	393 (0.7)	22,619 (30)	27 (1)	366 (0.7)	<0.01
TVR within 1 year *	2256 (5)	12,099 (22)	71 (5)	2256 (5)	0.16

Data are presented as number (%) or mean (±SD). PCI, percutaneous coronary intervention; CABG, coronary bypass grafting; eGFR, estimated glomerular filtration rate; LVEF, left ventricular ejection fraction; STEMI, ST-segment elevation myocardial infarction; NSTEMI, non-ST-segment elevation myocardial infarction; LAD, left anterior descending coronary artery; MI, myocardial infarction. * Only calculated for patients with completed 1-year follow-up (intervention year 2015, 2016, and 2017; *n* = 54,566).

**Table 2:** Results of univariable Cox regression analysis and multivariable model to predict mortality for patients with cardiogenic shock (*n* = 3028).

	HR	Univariable	*p*-Value	HR	Multivariable	*p*-Value
		95% CI			95% CI	
Age (years)	1.03	1.02–1.03	<0.001	1.02	1.02–1.03	<0.001
Male	0.13	0.77–0.97	0.01	0.97	0.84–1.11	0.62
Diabetes mellitus	1.57	1.38–1.80	<0.001	1.25	1.08–1.45	<0.01
Multivessel disease	1.52	1.35–1.71	<0.001	1.22	1.06–1.39	<0.01
CTO	1.29	0.96–1.74	0.09	1.16	0.82–1.64	0.39
Prior MI	1.36	1.18–1.56	<0.001	1.24	1.06–1.45	<0.01
Prior CABG	1.32	1.05–1.65	0.02	0.93	0.72–1.21	0.59
eGFR (mL/min/1.73 m^2^)	0.98	0.98–0.98	<0.001	0.98	0.98–0.99	<0.001
OHCA	1.44	1.29–1.60	<0.001	1.71	1.50–1.94	<0.001
STEMI	0.79	0.67–0.93	<0.01	0.88	0.74–1.06	0.18
PCI center	0.95	0.84–1.07	0.41			
Intervention year						
2015	1.14	0.97–1.32	0.11	1.14	0.95–1.35	0.15
2016	1.19	1.02–1.40	0.03	1.08	0.91–1.30	0.38
2017	1.08	0.93–1.26	0.33	1.18	0.99–1.42	0.07

Variables with *p* < 0.10 in the univariable analysis were included in the multivariable model. STEMI vs. NSTEMI; PCI center vs. heart center; reference intervention year was 2018. CTO, chronic total occlusion; MI, myocardial infarction; CABG, coronary bypass grafting; eGFR, estimated glomerular filtration rate; STEMI, ST-segment elevation myocardial infarction; NSTEMI, non-ST-segment elevation myocardial infarction; PCI, percutaneous coronary intervention.

## Data Availability

The data presented in this study are collected through the nationwide registry Netherlands Heart Registration and are not openly available. Data may be provided upon request.

## Supplementary Materials

The following are available online at https://www.mdpi.com/article/10.3390/jcm10102047/s1, Figure S1: The ROC curve for classification according to the multivariable model, Figure S2: Kaplan–Meier curves per age category, Figure S3: Kaplan–Meier curves according to renal function, Figure S4: Kaplan–Meier curves for patients with and without OHCA, Table S1: Results of Cox regression analysis excluding patients who died before or at 30-days.

## Appendix A

The following physicians are the members of PCI Registration Committee of the NHR. They represent the hospitals that have provided the PCI data for this study.

G.		Amoroso	OLVG
E.K.		Arkenbout	Tergooi
S.		Aydin	VieCuri Medisch Centrum
J.		Brouwer	Medisch Centrum Leeuwarden
C.		Camaro	RadboudUMC
J.		Daemen	Erasmus Medisch Centrum
P.		Danse	Rijnstate
S.F.		de la Fuente	Jeroen Bosch Ziekenhuis
M.	van der	Ent	Maasstad Ziekenhuis
R.		Erdem	ZorgSaam
P.	den	Heijer	Amphia
J.P.S.		Henriques	Amsterdam UMC, locatie AMC
A.W.J.	van ‘t	Hof	Zuyderland MC
A.W.J.	van ‘t	Hof	Academisch Ziekenhuis Maastricht
I.		Karalis	Leids Universitair Medisch Centrum
A.		Kraaijeveld	UMC Utrecht
J.P.	van	Kuijk	Sint Antonius Ziekenhuis
E.		Lipsic	Universitair Medisch Centrum Groningen
M.		Magro	Elisabeth-TweeSteden Ziekenhuis
K.M.J.		Marques	Amsterdam UMC, locatie VUmc
T.		Oude Ophuis	Canisius Wilhelmina Ziekenhuis
J.	van	Ramshorst	Noordwest Ziekenhuisgroep
V.		Roolvink	Isala
W.T.		Ruifrok	Treant Zorggroep
M.		Scholte	Albert Schweitzer Ziekenhuis
C.E.		Schotborgh	Haga Ziekenhuis
B.J.		Sorgdrager	Haaglanden Medisch Centrum
F.		Spano	Meander Medisch Centrum
M.G.		Stoel	Medisch Spectrum Twente
K.		Teeuwen	Catharina Ziekenhuis
