# Supplementary material for: Outcome and Predictors for Mortality in Patients with Cardiogenic Shock: A Dutch Nationwide Registry-Based Study of 75,407 Patients with Acute Coronary Syndrome Treated by PCI
Source: J Clin Med. 2021 May 11;10(10):2047. doi: 10.3390/jcm10102047 (PMC8151113; doi:10.3390/jcm10102047)
Supplement: Supplementary file 1 [file jcm-10-02047-s001.zip › jcm-1120072-supplementary.pdf]

SUPPLEMENTARY MATERIALS

Figure S1. The ROC curve for classification according to the multivariable model for predicting mortality in patients with cardiogenic shock, AUC = 0.73.

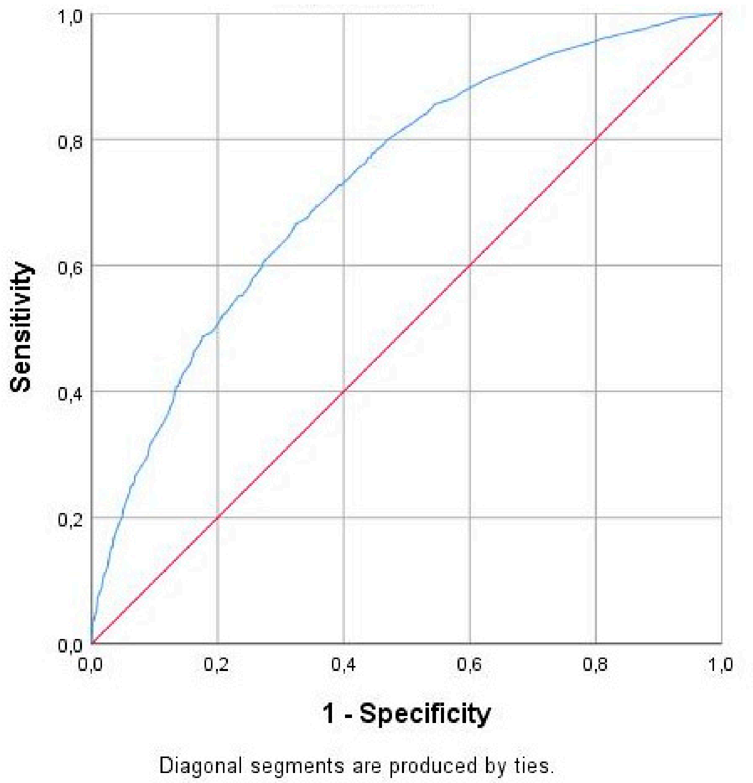

Table S1. Results of univariable Cox regression analysis and multivariable model to predict mortality for patients with cardiogenic shock excluding patients who died before or at 30-days (N=1,916).

	**Univariable**			**Multivariable**		
	HR	95% CI	p-value	HR	95% CI	p-value
Age (years)	1.05	1.04-1.07	<0.001	1.04	1.02-1.05	**<0.001**
Male	0.82	0.61-1.09	0.17			
Diabetes mellitus	2.28	1.68-3.09	<0.001	1.66	1.19-2.31	**<0.01**
Multivessel disease	2.17	1.61-2.94	<0.001	1.47	1.06-2.04	**0.02**
CTO	1.03	0.42-2.49	0.95			
Prior MI	2.61	1.94-3.52	<0.001	1.95	1.40-2.71	**<0.001**
Prior CABG	2.47	1.59-3.84	<0.001	1.17	0.71-1.91	0.54
eGFR (ml/min/1.73 m^2)	0.98	0.98-0.99	<0.001	0.99	0.99-1.00	**0.02**
OHCA	0.70	0.52-0.93	0.02	0.92	0.67-1.26	0.61
STEMI	0.57	0.39-0.84	<0.01	0.78	0.52-1.18	0.23
PCI center	0.88	0.64-1.20	0.40			
Intervention year						
2015	1.00	0.64-1.55	0.98			
2016	1.27	0.82-1.97	0.28			
2017	1.10	0.71-1.71	0.67			

Variables with p<0.10 in the univariable analysis were included in the multivariable model. STEMI vs NSTEMI; PCI center vs heart center; reference intervention year was 2018. CTO, chronic total occlusion; MI, myocardial infarction; CABG, coronary bypass grafting; eGFR, estimated glomerular filtration rate; STEMI, ST-segment elevation myocardial infarction; NSTEMI, non-ST-segment elevation myocardial infarction; PCI, percutaneous coronary intervention.

Figure S2. Kaplan Meier curves showing survival of cardiogenic shock patients treated by PCI (n=3,082) per age category, log-rank p<0.001.

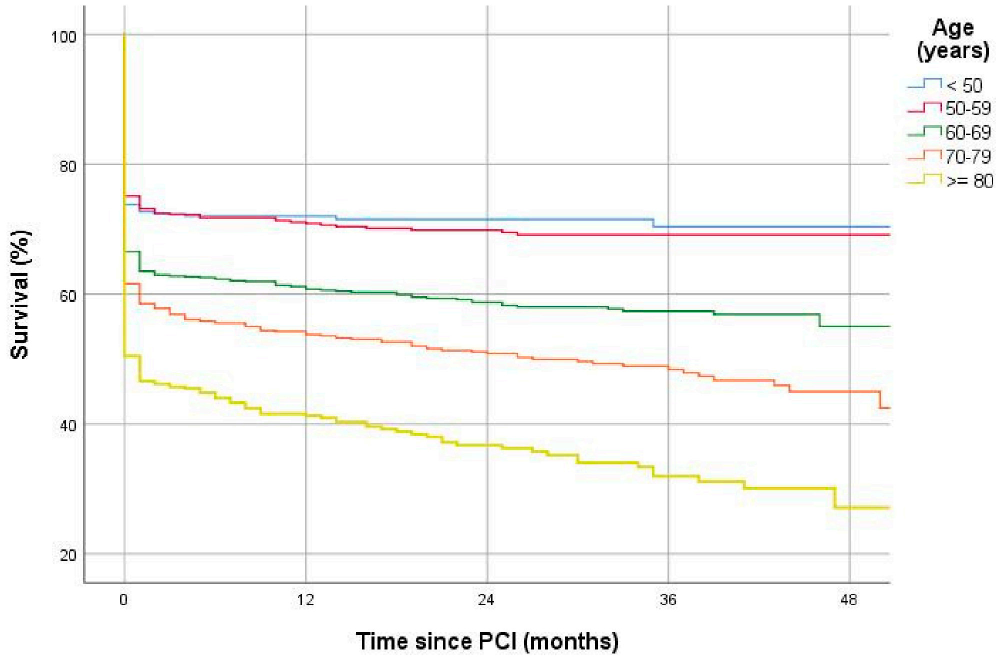

**Age**	**No. at Risk**				
**<50**	294	160	101	58	15
**50-59**	590	319	201	126	46
**60-69**	864	409	265	142	49
**70-79**	794	336	202	102	35
**>=80**	454	139	82	44	8

Figure S3. Kaplan Meier curves showing survival of cardiogenic shock patients treated by PCI (n=3,082) according to renal function, log-rank p<0.001.

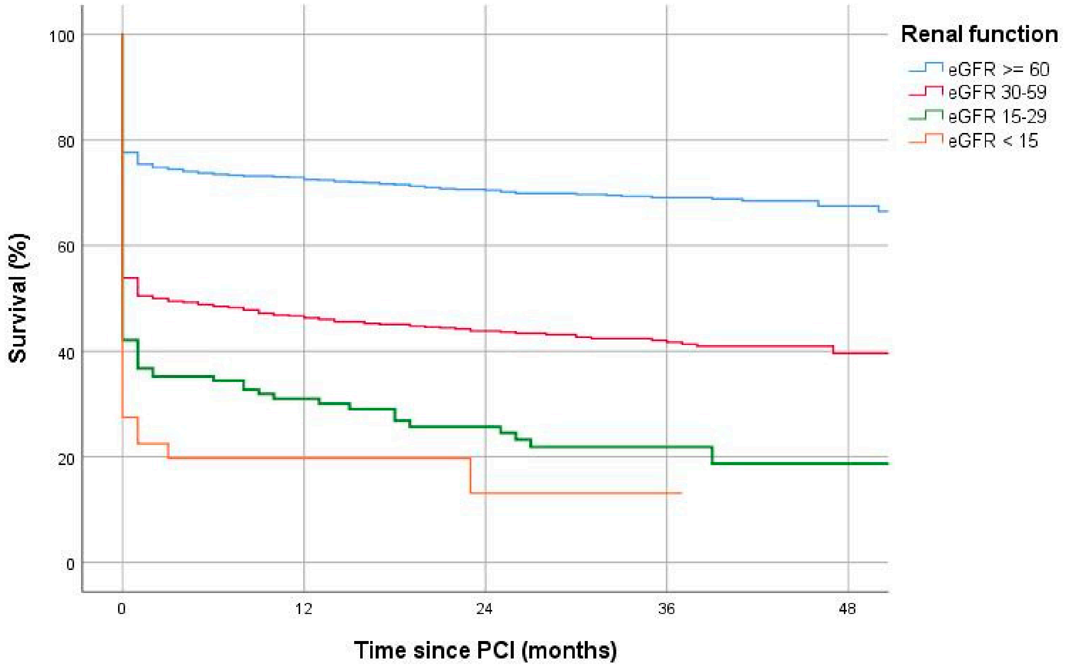

**eGFR**	**No. at Risk**				
**>=60**	1467	820	515	297	111
**30-59**	991	347	225	120	27
**15-29**	133	34	22	9	4
**<15**	40	6	2	1	0

Figure S4. Kaplan Meier curves showing survival of cardiogenic shock patients treated by PCI (n=3,082) for patients with and without OHCA, log-rank p<0.001.

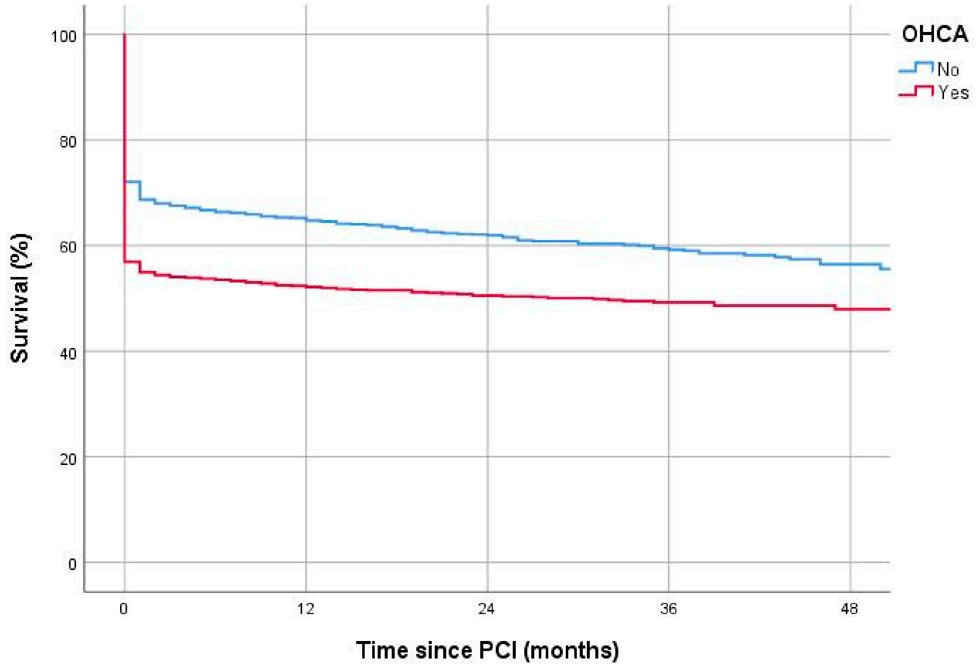

OHCA	No. at Risk				
**No**	1635	818	483	262	97
**Yes**	1355	542	365	208	56

=
